# A Formaldehyde Sensor Based on Molecularly-Imprinted Polymer on a TiO_2_ Nanotube Array

**DOI:** 10.3390/s17040675

**Published:** 2017-03-24

**Authors:** Xiaohui Tang, Jean-Pierre Raskin, Driss Lahem, Arnaud Krumpmann, André Decroly, Marc Debliquy

**Affiliations:** 1 ICTEAM, Université Catholique de Louvain (UCL), Place du Levant, 3, 1348 Louvain-la-Neuve, Belgium; jean-pierre.Raskin@uclouvain.be; 2 Department of Materials Science, Materia Nova ASBL, 7000 Mons, Belgium; driss.lahem@materianova.be; 3 Materials Science Department, University of Mons, 7000 Mons, Belgium; arnaud.krumpmann@umons.ac.be (A.K.); andre.decroly@umons.ac.be (A.D.); marc.debliquy@umons.ac.be (M.D.)

**Keywords:** formaldehyde sensor, molecularly-imprinted polypyrrole, titanium dioxide nanotube array, humidity influence

## Abstract

Today, significant attention has been brought to the development of sensitive, specific, cheap, and reliable sensors for real-time monitoring. Molecular imprinting technology is a versatile and promising technology for practical applications in many areas, particularly chemical sensors. Here, we present a chemical sensor for detecting formaldehyde, a toxic common indoor pollutant gas. Polypyrrole-based molecularly-imprinted polymer (PPy-based MIP) is employed as the sensing recognition layer and synthesized on a titanium dioxide nanotube array (TiO_2_-NTA) for increasing its surface-to-volume ratio, thereby improving the sensor performance. Our sensor selectively detects formaldehyde in the parts per million (ppm) range at room temperature. It also shows a long-term stability and small fluctuation to humidity variations. These are attributed to the thin fishnet-like structure of the PPy-based MIP on the highly-ordered and vertically-aligned TiO_2_-NTA.

## 1. Introduction

Formaldehyde (HCHO) is the most common indoor pollutant gas [1,2]. It is considered toxic and carcinogenic. The main sources of HCHO are essentially incomplete combustion and emission from paints, linoleums, varnishes, upholsteries, timbers, and plastic products. The World Health Organization (WHO) set a 30-min threshold limit of 0.08 ppm [3] and the US National Institute for Occupational Safety and Health (NIOSH) established a maximum long-term exposure limit of 0.016 ppm [4]. These two numbers point out the ultimate goal for the HCHO sensor development. Many detection methods have been studied, for example, spectrophotometer [5], chromatography [6], fluorescence [7], piezoresistance [8], polarography [9], quartz crystal microbalance [10], microelectromechanical systems [11], metal oxide films [12], nanowires [13], nanotubes [14], and conductive polymers [15]. Recently, more attention has been paid to the development of sensitive, specific, cheap, and reliable sensors with real-time monitoring.

Molecular imprinting technology (MIT) is, today, a versatile and promising technology for practical applications in many areas, such as antibody receptors [16,17], protein separation [18,19,20], pollutant determination [21], purification science [22], catalysis [23,24], drug delivery [25] and, particularly, chemical sensor [26,27,28]. MIT is based on the synthesis of specific polymers able to recognize biological and chemical molecules [29]. In brief, the principle is that functional monomers, having an affinity for a kind of given template (target) molecules, are polymerized around the template to form a polymer network. After the polymerization process, the template molecules are removed from the polymer, leaving specific recognition sites complementary in shape, size, and chemical functionality to the target molecules [30]. The resultant polymer can selectively rebind the target molecules. Molecularly-imprinted polymers (MIPs) usually demonstrate the following advantages: high selectivity and affinity for target molecules; physical robustness, strength, and resistance to temperature and pressure; chemical inertness towards acids, bases, metal ions, and organic solvents; and long-term stability. MIPs have been exploited as sensitive elements in chemical sensors [31], quartz crystal microbalances [32], and chromatography analyzers [33].

Generally, the MIP layers have a low surface-to-volume ratio; thus, the majority of the active sensing component is embedded in the bulk, which inevitably influences the efficiency and sensitivity. To reduce the diffusion pathway of target molecules, Hussain and co-workers [34] developed a MIP nanoparticle sensor for HCHO detection. They obtained a detection limit of 0.5 ppm. However, their sensor needs further functionalization for sensing in real-life conditions of 50% relative humidity. Moreover, the authors [35,36,37] deposited a uniform and ultrathin MIP layer on a porous material, which provides improved sensitivity. Among various candidates of porous materials, titanium dioxide nanotube arrays (TiO_2_-NTA) are very interesting owing to their highly porous structure and low synthesis cost. We present a HCHO sensor in which polypyrrole-based molecularly-imprinted polymer (PPy-based MIP) is employed as the sensing recognition layer. For improving the porosity, the surface-to-volume ratio, and the sensor performance, the PPy-based MIP is synthesized on TiO_2_-NTA. Hereafter, our sensor is referred to as the PPy-based MIP/TiO_2_-NTA sensor.

## 2. Materials and Methods

### 2.1. Synthesis of Polypyrrole-Based Molecularly-Imprinted Polymer

**Figure 1:**
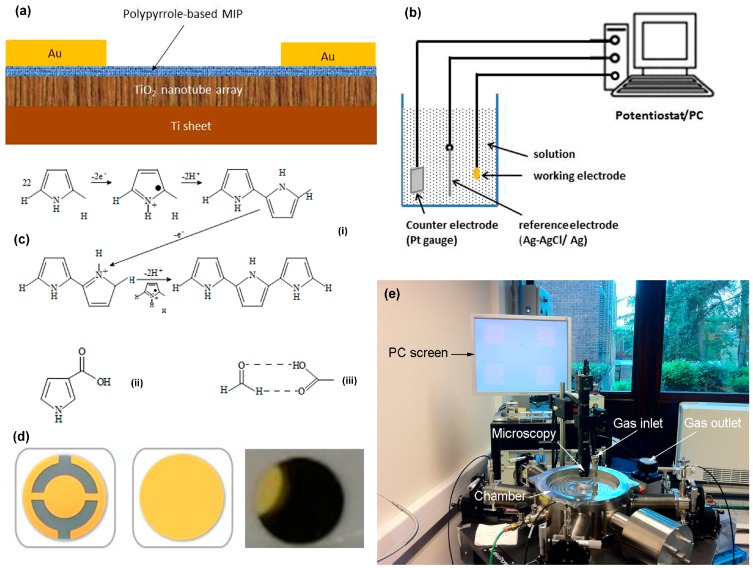
(**a**) Schematic cross-section of the polypyrrole-based MIP/TiO_2_-NTA sensor, showing the molecularly-imprinted polypyrrole synthesized on TiO_2_ nanotube array; (**b**) schematic illustration of the electropolymerization system; (**c**) chemical scheme illustrations ((**i**) polymerization mechanism of polypyrrole, (**ii**) structure of pyrrole-carboxylic, and (**iii**) specific bond of pyrrole-carboxylic with formaldehyde); (**d**) quartz crystal wafers with circular gold electrodes and with the polypyrrole-based MIP layer; and (**e**) image of the gas sensing setup.

Figure 1a schematically illustrates the cross-section of the PPy-based MIP/TiO_2_-NTA sensor. Its fabrication process includes three main steps: TiO_2_-NTA preparation, PPy-based MIP synthesis, and electrode patterning. The TiO_2_-NTA is first prepared on a titanium (Ti) sheet with an area of 2.5 cm × 2.5 cm and a thickness of 0.5 mm by anodic oxidation. The Ti sheet is cleaned with isopropanol and treated with 1 M nitric acid for 30 min. For the anodization, the Ti sheet is used as the anode electrode and platinum (Pt) as the cathode electrode in an electrochemical bath filled with 500 mL ethylene glycol, 10 mL deionized water, and 1.7 g NH_4_F. A constant voltage of 40 V is biased at the two electrodes for four hours. As a result, a highly-ordered and vertically-aligned TiO_2_-NTA is formed on top of the Ti sheet. The sample is then sonicated in a deionized water bath for 30 s to remove the eventual residue blocking the pores. After washing with deionized water and drying, the TiO_2_-NTA/Ti sheet is annealed for 2 h at 475 °C in air.

There are various techniques for synthesizing the MIP layers: electropolymerization [38], spin coating [39], and laser deposition [40]. In this work, we synthesize the PPy-based MIP layer on the TiO_2_-NTA/Ti sheet by direct electropolymerization [41]. As shown in Figure 1b, the electropolymerization is carried out in a solution of 50 mL acetonitrile, 0.614 g sodium perchlorate, 0.17 g sodium dodecylsulfate, 0.335 g pyrrole, 55 mg pyrrole-3 carboxylic acid, and 10 mL HCHO (37% in water). The TiO_2_-NTA/Ti sheet is used as the working electrode. The counter and reference electrodes are made of Pt and Ag/AgCl, respectively. The electropolymerization mechanism is sketched in Figure 1c(i). Pyrrole-3 carboxylic acid is added because it can build hydrogen bonds with formaldehyde as shown in Figure 1c(ii). We investigated the influence of the pyrrole-3-carboxylic acid/pyrrole ratio on the PPy-based MIP performance. The results of the mass measurements indicate that the performance improvement increased with the amount of pyrrole-3-carboxylic acid. When the ratio is larger than 10%, the improvement becomes less obvious. On the other hand, we considered the high cost of pyrrole-3-carboxylic acid and then optimized the amount of 55 mg in our work. Pulse voltages of 2 V for 0.1 s and −0.3 V for 0.04 s are applied to the electrodes. After 1000 cycles, a dark PPy-based MIP layer is synthesized on top of the TiO_2_-NTA/Ti sheet. The template molecules (HCHO) are removed from the PPy-based MIP layer by dipping in a mixture of 1/3 acetic acid and 2/3 methanol for 8 h. It is worth noting that there are two reactions in competition during the PPy-based MIP synthesis: electropolymerization and formaldehyde oxidation. However, we checked the current of the formaldehyde oxidation (same solution without pyrrole) and found that it is much smaller than that of the electropolymerization in the used potential range. Therefore, the electropolymerization dominates the competition. For comparison, the non-imprinted PPy layer (called NIP) is also synthesized in the same conditions without template molecules. Finally, gold (Au) electrodes with an area of 0.8 mm × 0.8 mm and a thickness of 50 nm are patterned on top of the PPy-based MIP layer by using a hard masker. To increase the adhesion, a titanium (Ti) layer of 5 nm is deposited between Au and the PPy-based MIP layer.

### 2.2. Physical Characterization System and Electric Sensing Setup

Mass changes are measured by using quartz crystal microbalance (QCM) [42], which uses the change in the frequency of a quartz resonator to measure the mass of a film adherently deposited on the resonator surface. The basic design of the QCM is a thin quartz crystal wafer with circular gold electrodes deposited on both sides. The back side of the QCM wafer is protected to avoid depositing. The PPy-based MIP and NIP films are directly deposited on the QCM wafers by electropolymerization, as described above. The uniformity and adhesion of the obtained films is quite good (Figure 1d). The QCM wafer with the PPy-based MIP is then mounted in a homemade gas chamber and connected to an oscillating circuit for measuring the resonance frequency.

In order to perform the electrical sensing measurements, a PPy-based MIP/TiO_2_-NTA sensor is placed in a sealed chamber with gas inlet and outlet ports as shown in Figure 1e. A microscope is installed on top window of the sealed chamber to observe the electrodes on the sensor surface through the computer’s screen. To eliminate the contact and cable resistances, the measurements are carried out by the four-point probes method [43]. Hereafter, we refer to the sensor conductance recorded at a constant current of 1 mA, at atmospheric pressure with a temperature of 22 °C, and a relative humidity of 50%, unless otherwise noted. HCHO and air are used as target and carrier gases, respectively. The desired HCHO concentration is obtained by dilution of 100 ppm of HCHO in air. 

## 3. Results

### 3.1. Physical Characterization of Materials

**Figure 2:**
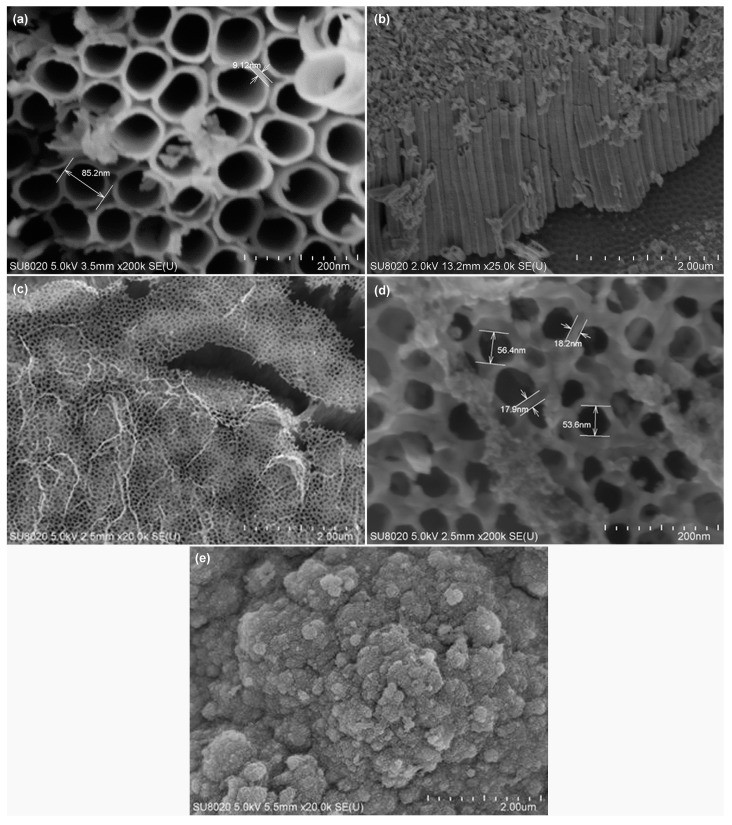
Scanning electron microscope images of (**a**) top view of TiO_2_ nanotube array, (**b**) cross-section view of TiO_2_ nanotube array, (**c**) thin layer of molecularly-imprinted polypyrrole synthesized on a TiO_2_ nanotube array, (**d**) zoom of (**c**); and (**e**) thick polypyrrole film on the flat substrate.

The morphology of the TiO_2_-NTA and PPy-based MIP layer is characterized by scanning electron microscopy (SEM). Figure 2a presents the top-view SEM image of the TiO_2_-NTA before synthesizing the PPy-based MIP layer. TiO_2_ nanotubes have an average diameter of 85 nm and an average wall thickness of 10 nm. Figure 2b shows the cross-section SEM image of the TiO_2_-NTA. TiO_2_ nanotube has a length of about 2 µm. Figure 2c,d exhibit the top-view SEM images for the PPy-based MIP layer on the TiO_2_-NTA. The PPY-based MIP layer has a fishnet-like structure and its thickness is 20 nm. Figure 2e provides the top-view SEM image for a PPy-based MIP film synthesized on a flat substrate. It has a typical cauliflower structure with a thickness of 1.5 µm.

### 3.2. Chemical Compositions of Materials

**Figure 3:**
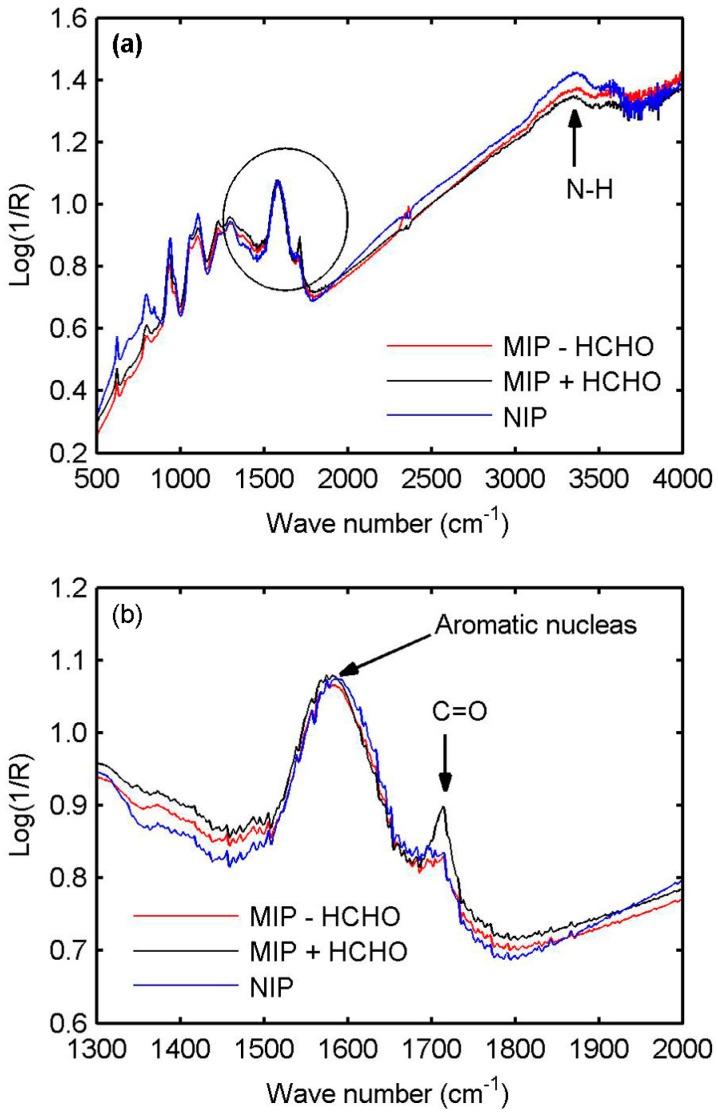
FTIR spectra of (**a**) molecularly-imprinted polypyrrole films synthesized without the HCHO template, with HCHO template, and after removing the HCHO template; and (**b**) zoom on the insert circle in (**a**).

To identify the compositions and chemical bonds of the MIP and NIP films, Fourier Transform Infrared Spectroscopy (FTIR) analysis is carried out on NIP and MIP films with a thickness of 1.5 µm, deposited on gold QCM substrates to obtain a good signal in reflection. Figure 3a presents the FTIR spectra before (immediately after the deposition and a rapid rinsing with ethanol) and after removing the HCHO molecules, respectively. The FTIR spectrum of the NIP film is also shown in the figure for the comparison. The three spectra are similar. The main difference is the appearance of a peak (located at 1715 cm^−1^) for the MIP film before removing the HCHO molecules. This can be clearly observed in Figure 3b, which zooms on the insert circle in Figure 3a. After removing the HCHO molecules, the peak disappears and the related spectrum is very similar to that of the NIP film. This peak corresponds to the double bond between C and O, which is the evidence for the HCHO molecules existing within the MIP film. The peak (located between 1500 and 1650 cm^−1^) is related to the aromatic nucleus, while the other one between 3000 and 3500 cm^−1^ is the characteristic peak of the N–H bond of PPy. Both peaks indicate the presence of the pyrrole nucleus. All of the chemical information confirms that the synthesized PPy-based MIP film by electropolymerization contains PPy and HCHO molecules. Although the FTIR spectra are obtained from the thick PPy-based MIP film (1.5 µm), they can be extrapolated to the thin PPy-based MIP layer on TiO_2_ since the experimental conditions of the film and layer are the same.

**Figure 4:**
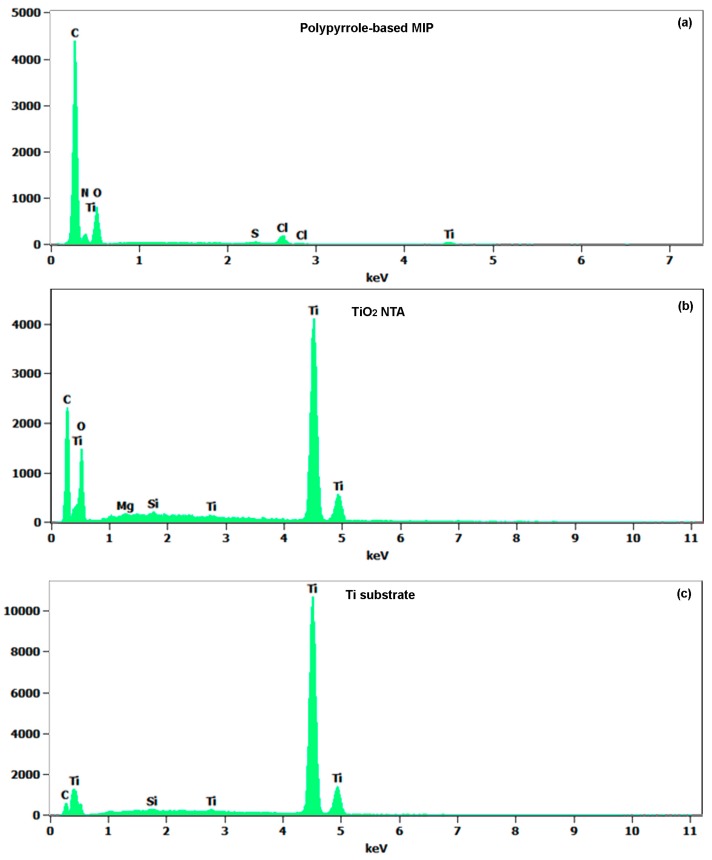
Energy dispersive X-ray spectroscopy spectra of (**a**) molecularly-imprinted polypyrrole; (**b**) TiO_2_ nanotube array; and (**c**) Ti sheet substrate.

Energy dispersive X-ray spectroscopy (EDX) analysis is performed on a PPy-based MIP/TiO_2_-NTA/Ti stack and the results are shown in Figure 4. The atomic concentrations of the main elements on each layer are summarized in Table 1. The related errors are also given in the table. The analysis depth is quite large so that the electron beam reaches the Ti substrate. The Ti atomic concentrations in the PPy-based MIP layer, TiO_2_-NTA, and Ti substrate are 1.1, 26.9, and 91.1 at %, respectively. Moreover, the elemental composition of the TiO_2_-NTA can be estimated from the atomic ratio [O/Ti] = 1.78 in the table. We now focus on the C atomic concentration of each layer. An increase of the C concentration is observed in TiO_2_ film (24.8 at %), most likely originating from the PPy-based MIP layer residues coated on the TiO_2_-NTA wall and bottom. Figure 4a exhibits the C peak (50.6 at %) and the N peak (17 at %), which correspond to the PPy-based MIP layer. Only a small amount of Ti (1.1 at %) is found in the PPy-based MIP layer. This fact indicates Ti dispersion or distribution into the PPy-based MIP layer is small during the PPy-based MIP synthesis.

### 3.3. Specific Adsorption of PPy-Based MIP Film

**Figure 5:**
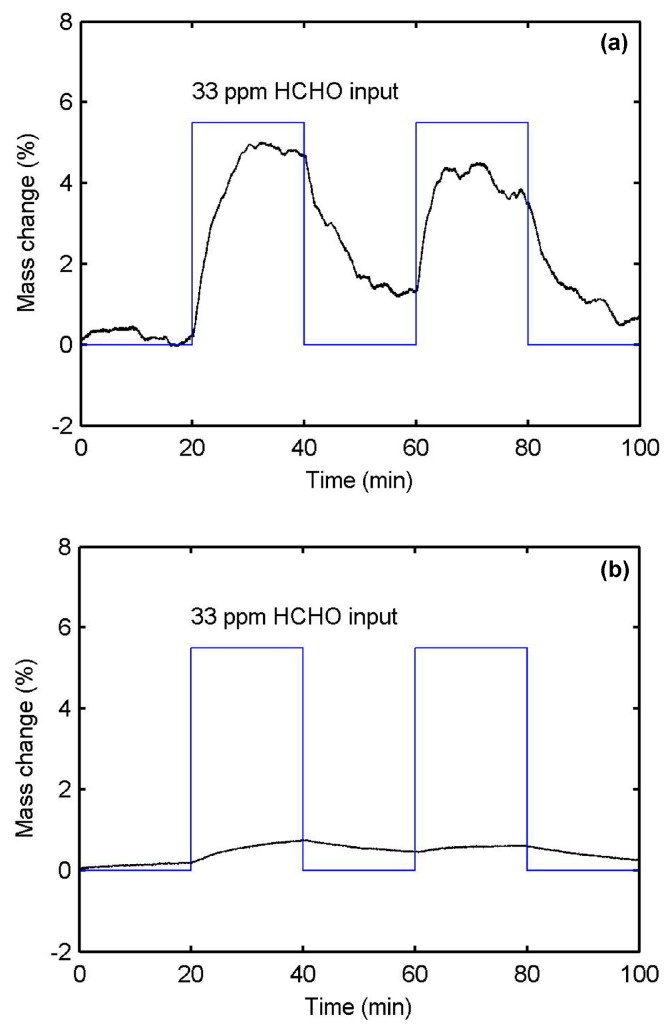
Mass changes for molecularly-imprinted polypyrrole films synthesized on quartz crystal wafers (**a**) with and (**b**) without HCHO template, showing uptake during 33 ppm HCHO injections at 22 °C in 50% RH air.

To compare the affinity of the PPy-based MIP and NIP films to HCHO molecules, both films are synthesized by electropolymerization on QCM wafers under the same experimental conditions for mass measurements. The obtained films present the cauliflower structure as shown in Figure 2e due to the absence of the TiO_2_-NTA supporting substrate. According to the Sauerbrey equation, the resonance frequency change of the QCM wafer Δf is defined by $\Delta f=\frac{-2\Delta mf_{o}^{2}}{A\sqrt{\rho_{q}{µ}_{q}}}\;\left(\mathrm{Hz}\right)$, where Δm (g) is the mass change, f_0_ (Hz) is the initial resonant frequency, which is 6 MHz in our measurements, A (cm^2^) is the piezoelectrically active crystal area, and ρ_q_ and µ_q_ are the density and Shear modulus of quartz, respectively. The frequency of the QCM is measured before the deposition of the polymer (bare QCM) and after deposition and template removal to obtain the film initial mass. The gas adsorption measurements can then be expressed in terms of relative mass change ((mass with gas – initial mass)/initial mass in %). In Figure 5a, we observe that the PPy-based MIP film shows a rapid and reversible mass change of 5% to the 33 ppm HCHO injections. On the contrary, the PPy-based NIP film produces a mass change less than 1% as shown in Figure 5b. These confirm the PPy-based MIP film has much better affinity to HCHO molecules than the PPy-based NIP film. The recovery mass change means that the adsorption process of HCHO is reversible at room temperature.

**Figure 6:**
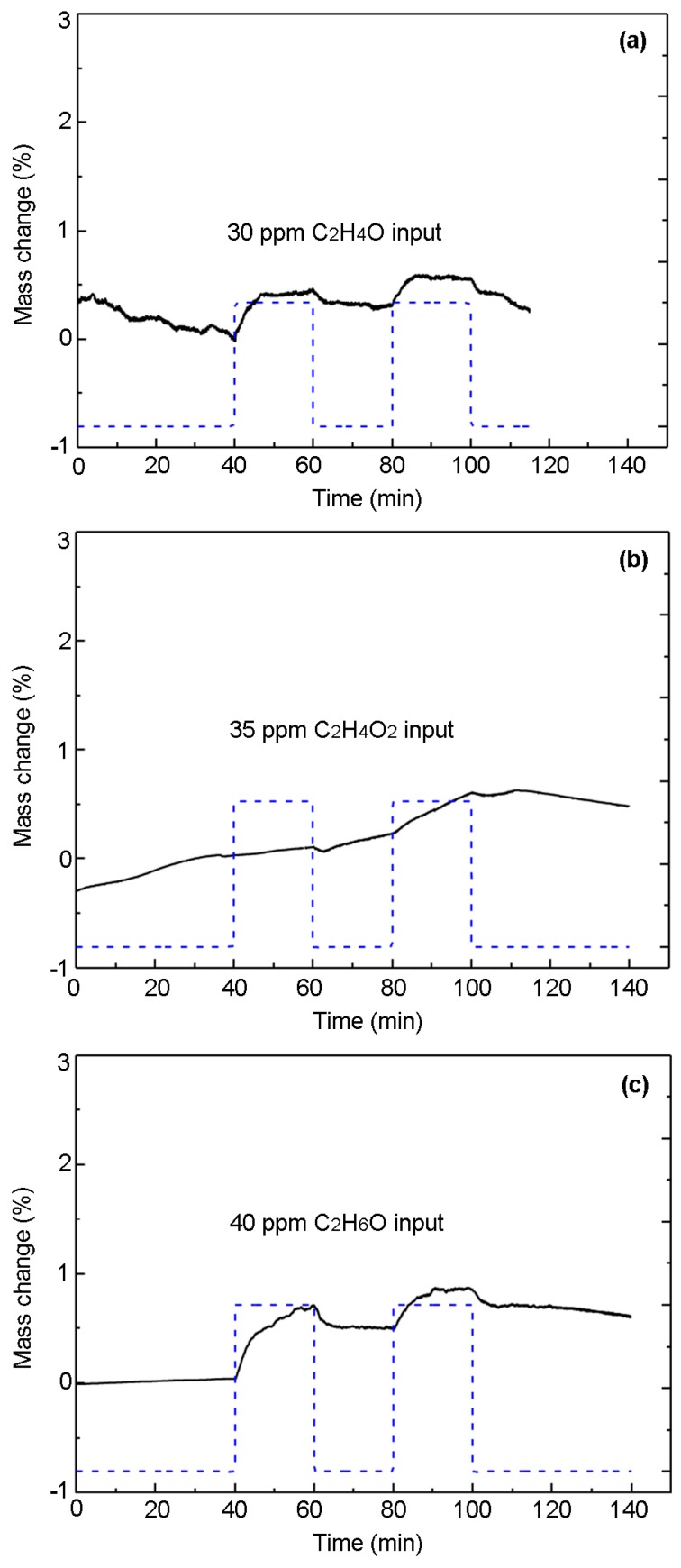
Mass changes for molecularly imprinted polypyrrole films synthesized on quartz crystal wafers, uptake during (**a**) 30 ppm C_2_H_4_O; (**b**) 35 ppm C_2_H_4_O_2_; and (**c**) 40 ppm C_2_H_6_O at 22 °C in 50% RH air.

We evaluate the selectivity of the PPy-based MIP film to the interference gases containing the CHO group. To this end, the PPy-based MIP film synthesized on QCM wafer is exposed to acetaldehyde (C_2_H_4_O), acetic acid (C_2_H_4_O_2_), and ethanol (C_2_H_6_O), respectively. It can be seen from Figure 6 that the mass changes are much smaller (less than 1%) than that in Figure 5a, corresponding to HCHO. This is due to the fact that the recognition sites formed on the surface of the PPy-based MIP film cannot adsorb the interference gases [44]. This embodies the good selectivity of the PPy-based MIP film to HCHO molecules. 

### 3.4. Electrical Sensing Measurement Results

**Figure 7:**
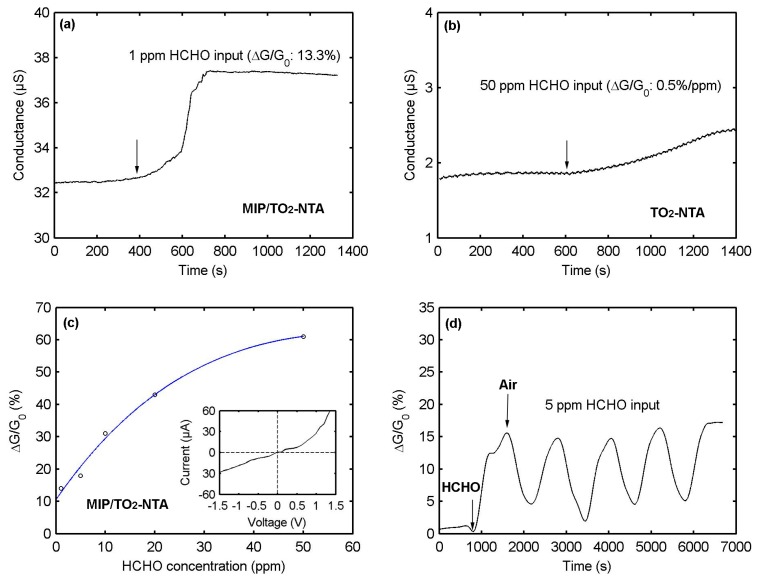
Conductance behaviors of (**a**) polypyrrole-based MIP/TiO_2_-NTA sensor for detecting 1 ppm HCHO; (**b**) TiO_2_ nanotube array for detecting 50 ppm HCHO; (**c**) dependence between conductance responses and HCHO concentrations for the polypyrrole-based MIP/TiO_2_-NTA sensor; and (**d**) conductance responses after the polypyrrole-based MIP/TiO_2_-NTA sensor exposed to five cycles of 5 ppm HCHO.

The relative change of conductance is measured to investigate the sensor conductance response, which is defined as ∆G/G_0_ = (G − G_0_)/G_0_, where G_0_ and G are, respectively, the conductance of the sensor before and after exposure to the target gas. As can be seen in Figure 7a, the injection of 1 ppm HCHO causes a conductance response of 13% for a typical PPy-based MIP/TiO_2_-NTA sensor. In the sensor fabrication, the TiO_2_-NTA supporting substrate is used for increasing the porosity of the PPy-based MIP. To check if the conductance response is really due to the PPy-based MIP layer, rather than the TiO_2_-NTA substrate, we directly deposited the Au electrodes on top of the TiO_2_-NTA substrate without the MIP. The sensing tests are carried out with a concentration of 50 ppm HCHO. A conductance increase of 0.5% per ppm is obtained from the TiO_2_-NTA substrate (Figure 7b). Though TiO_2_ material is sensitive for HCHO, it does not works at room temperature. Therefore, the observed conductance response in Figure 7a is not caused by the TiO_2_-NTA substrate. We believe that the conductance response of the sensor can mainly be attributed to the PPy-based MIP layer. Figure 7c shows the conductance responses as a function of the HCHO concentrations for the PPy-based MIP/TiO_2_-NTA sensor. The dependence between the conductance responses and the HCHO concentrations can be filled by an exponential function. The solid line is the fitting curve and the open circles are experimental data. We obtain a detection limit of 1 ppm, which is close to Hussain’s result (0.5 ppm). The insert in Figure 7c is the current-voltage characteristic of the sensor, which is not Ohmic behavior and caused by the Schottky barrier between Au electrodes and the PPy-based MIP layer. Figure 7d shows the conductance responses after the sensor is exposed to five cycles of 5 ppm HCHO. The HCHO exposure time is 10 min, followed by 10 min of purge with air (50% RH). The sensor has a repeatable response, suggesting good reproducibility. Moreover, the conductance response remains unchanged after one year, showing long-term stability. The response and recovery times of the sensor are estimated to be about 300 s.

**Figure 8:**
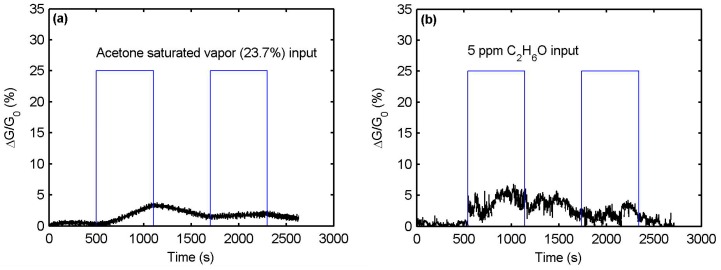
Polypyrrole-based MIP/TiO_2_-NTs sensor for detecting (**a**) acetone-saturated vapor (*‎*C_3_H_6_O, 23.7%) and (**b**) 5 ppm ethanol (C_2_H_6_O).

To investigate the sensor selectivity, acetone (‎C_3_H_6_O) and ethanol (C_2_H_6_O) are chosen as the interference gases since they contain the carbonyl functionality (C=O). The conductance response of the PPy-based MIP/TiO_2_-NTA sensor exposed to acetone saturated vapor in N_2_ is reported in Figure 8a. The sensor only produces a slight signal (3%) for acetone-saturated vapor (23.7% at 22 °C). Figure 8b reports the conductance response of the PPy-based MIP/TiO_2_-NTA sensor to 5 ppm ethanol. A visible conductance response is not observed except for electronic noise. The results in Section 3.3 have indicated that the PPy-based MIP film show a smaller absorption capacity to ethanol, acetaldehyde and acetic acid compared to formaldehyde. All the facts confirm that our sensor has a good selectivity to HCHO compared to the interference gases: acetone, ethanol, acetaldehyde, and acetic acid.

**Figure 9:**
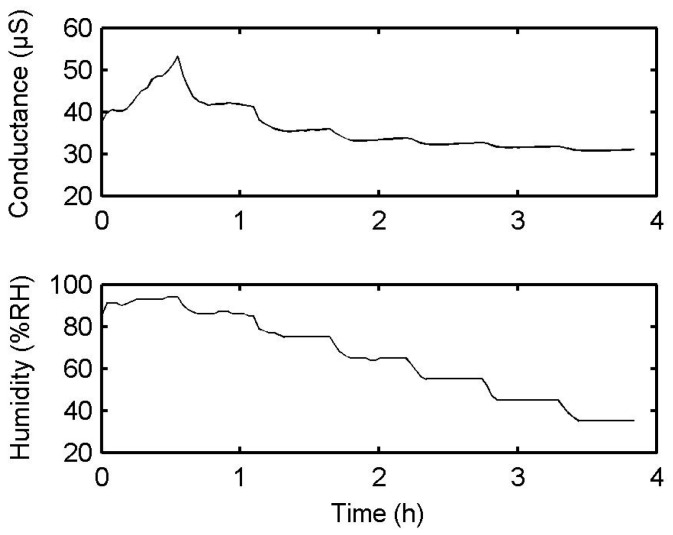
Conductance behavior of the polypyrrole-based MIP/TiO_2_-NTA sensor for the humidity range from 35% to 95% at 22 °C.

We tested the influence of humidity variations on the PPy-based MIP film itself. As shown in Figure 9, the conductance of the PPy-based MIP film increases from 30.74 to 53.25 µS within the humidity range from 35% to 95%. Specifically, a humidity change of 10% brings about a conductance change of 3.75%, on average. Obviously, the non-specific water molecules adsorb onto the film surface and they compete with the selective incorporation of HCHO molecules. The water molecules play the same role as HCHO molecules in increasing the film conductance, donating electrons into the PPy-based MIP film. However, the conductance change (3.75%) caused by water molecules is smaller than that (13%) of 1 ppm HCHO. 

Finally, we summarize the recently published HCHO sensors based on the polymers, operating at room temperature in Table 2. The key parameters of our sensor are also listed in the table for the comparison. The research on the polymer-based sensors is relatively immature. The sensor detection limits are at ppm levels. Most of the sensors have long response and recovery times.

## 4. Discussion

**Figure 10:**
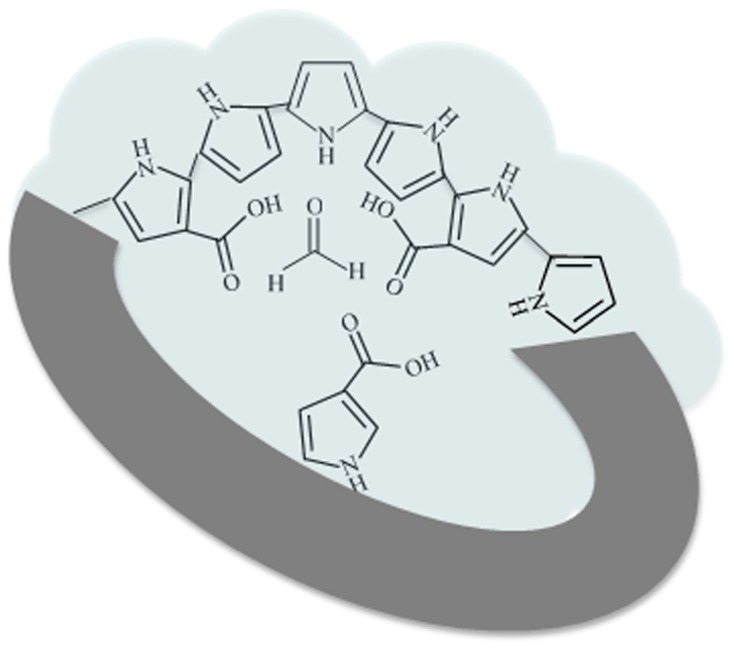
Chemical scheme illustration for the cavities on the polypyrrole-based MIP layer to detect formaldehyde.

The sensing mechanism of the PPy-based MIP layer can be explained and discussed as follows. The as-synthesized PPy-based MIP layer acts as an intrinsic semiconductor which has a low conductance. The cavities imprinted on the PPy-based MIP layer can distinguish HCHO from other interference gases through molecular size and monomer functionalities (Figure 10). When the PPy-based MIP layer is exposed to HCHO, it specifically rebinds the HCHO molecules by the bond interactions shown in Figure 1c(iii). The bonded HCHO molecules donate electrons to the PPy-based MIP layer [55]. In this case, the intrinsic semiconductor is n-type doped, thereby its conductance is increased. Apart from that, the conjugation effect [56] and the swelling effect [57] of the PPy-based MIP layer may accelerate the electron transfer and increase the mobility, thereby increasing the conductance. One advantage of our sensor is its excellent selectivity due to small size of the HCHO molecule combined with its very distinct functionalities (C=O and C–H). It is well known that the HCHO molecule is very small compared with C_2_H_4_O, C_2_H_4_O_2_, C_2_H_6_O, and C_3_H_6_O molecules and, of course, larger molecules. The cavities imprinted by the HCHO template are not comparable with these interference molecules in size. Although the interference gases and HCHO molecules contain the same C=O bond, the other bond of HCHO (C–H) ensures it is specifically bonded on the PPy-based MIP layer. The other advantage of our sensor is its high sensitivity. This is due to the thin fishnet-like structure of the PPy-based MIP layer. Indeed, its short diffusion pathway and large volume-to-surface ratio allow the HCHO molecules to efficiently access recognition cavities on the PPy-based MIP layer. Moreover, our sensor shows an advantage of long-term stability.

## 5. Conclusions

This paper presents a novel sensor for formaldehyde detection. Molecularly-imprinted polypyrrole is employed as the recognition layer and formaldehyde molecules are chosen as the template. The polypyrrole-based molecular imprinted polymer (PPy-based MIP) is synthesized on a titanium dioxide nanotube array (TiO_2_-NTA) for increasing its surface-to-volume ratio. The resulting PPy-based MIP layer presents a fishnet-like structure with a thickness of 20 nm. This enhances the bonding capability of the PPy-based MIP layer and minimizes gas diffusion resistance, leading to a high response of 13% for 1 ppm formaldehyde at room temperature. Apart from that, our sensor exhibits a good selectivity towards formaldehyde compared with acetone, acetaldehyde, acetic acid, and ethanol. Importantly, our sensor has a stability of more than one year and rather good immunity to humidity. Our research is beneficial to pushing forward practical applications of HCHO sensors.

## Figures and Tables

**Table 1:** A summary of the atomic concentrations of main elements for the polypyrrole-based MIP/TiO_2_-NTA/Ti stack.

	*C-K*	*O-K*	*Mg-K*	*N-K*	*Si-K*	*S-K*	*Ti-K*
	*at %*	*at %*	*at %*	*at %*	*at %*	*at %*	*at %*
**PPy-MIP**	50.6 ± 0.7	29.6 ± 1.3		17 ± 2.8		0.3 ± 0.1	1.1 ± 0.2
**TiO_2_ NTA**	24.8 ± 0.5	47.8 ± 1.1	0.2 ± 0.1		0.2 ± 0.0		26.9 ± 0.3
**Ti substrate**	8.4 ± 0.5				1.5 ± 0.1		91.1 ± 0.8

**Table 2:** Summary of typical research results on polymer-based sensors for HCHO detection at room temperature.

Sensor Type	Sensing Material	Response Value	Response Time	Recovery Time	Detection Limit	Reference
QCM	PEI/PVA	0.5 Hz/ppm			10 ppm	[45]
QCM	PEI/PS	1.5 Hz/3 ppm			3 ppm	[46]
QCM	PEI/PVA	0.8 Hz/1 ppm	120 s		1 ppm	[47]
QCM	MIP-NP	24 Hz/100 ppm	30 s		0.5 ppm	[34]
OFET	P_3_HT/Fe_2_O_3_	16%/100 ppm				[48]
OFET	P_3_HT/ZnO	20%/100 ppm				[49]
IDE	(PANi)xMoO_3_	8%/50 ppm				[50]
IDE	PANi/PEI	3.9%/1 ppm		20 s	1 ppm	[31]
IDE	PPy/EBSA	40%/500 ppm	300 s	>300 s		[51]
Chemiresistor	PPy/ZSI	4%/990 ppm	20 s			[52]
Chemiresistor	PMMA/RGO	30.5%/1000 ppm	150 s	180 s	100 ppm	[53]
Chemiresistor	PANi/LYS	15%/75 ppm			0.4 ppm	[54]
Chemiresistor	MIP/TiO_2_ NTA	13%/1 ppm	300 s	300 s	1 ppm	This work

QCM: quartz crystal microbalance, OFET: organic field-effect transistor, IDE: interdigitated electrodes, PEI: polyethyleneimine, P_3_HT: poly-3-hexylithiophene, PANi: polyaniline, PPy: polypyrrole, RGO: reduced graphene oxide. The rest of the abbreviations can be found in the related references.
